# Patient-Reported Outcomes on Quality of Life and Psychological Distress After Focal High-Intensity Focused Ultrasound for Prostate Cancer: Prospective Multicenter Study

**DOI:** 10.2196/91984

**Published:** 2026-06-15

**Authors:** Toni Franz, Cornelia Hartung, Roman Ganzer, Sigrun Holze, Jens-Uwe Stolzenburg

**Affiliations:** 1 Department of Urology, University Hospital Leipzig, Liebigstraße 20, Leipzig, Saxony, 04103, Germany, 49 03419717694; 2 Leipzig Network for Integrative Psychotherapy, Leipzig, Saxony, Germany; 3 Department of Urology, Asklepios Hospital Bad Tölz, Bad Tölz, Bavaria, Germany

**Keywords:** prostate cancer, focal HIFU, high-intensity focused ultrasound, hemiablation, quality of life, psychological distress, patient-reported outcomes

## Abstract

**Background:**

Focal high-intensity focused ultrasound (HIFU) is an emerging tissue-preserving treatment for localized prostate cancer (PCa) that aims to reduce functional impairment and psychological burden while maintaining oncological safety. Although its clinical use is increasing, prospective data on health-related quality of life (HRQoL) and psychological distress after focal hemiablation remain limited.

**Objective:**

This study aims to evaluate longitudinal HRQoL trajectories and psychological outcomes in patients eligible for active surveillance who underwent focal HIFU following shared decision-making.

**Methods:**

This prospective, multicenter, single-arm longitudinal study included men with unilateral, organ-confined PCa treated with focal HIFU hemiablation within the HEMI study (AP study protocol/reference number: 68/11, AUO). All patients had concordant multiparametric magnetic resonance imaging and biopsy findings, and underwent standardized unilateral HIFU. HRQoL was assessed at baseline and at 1, 3, 6, 9, and 12 months using the European Organization for Research and Treatment of Cancer Quality of Life Questionnaire—Core 30 (EORTC QLQ-C30, version 3.0). Psychological distress was measured with the Hospital Anxiety and Depression Scale. Longitudinal changes were analyzed using linear mixed-effects models with false discovery rate correction. In addition, univariate regression analyses were performed to assess associations between clinical and functional parameters, and HRQoL outcomes. Outcomes were compared with German and Norwegian normative populations, and clinical relevance was interpreted using Osoba criteria.

**Results:**

Fifty-four patients were included, of whom 51 (94.4%) completed 12-month follow-up (mean age 63.4, SD 8.3 y). Baseline HRQoL, functional status, and symptom burden were favorable. One month after hemi-HIFU, a statistically significant and clinically moderate decline in global health status was observed (mean 75 [SD 17.8] vs 63 [SD 17.9]; *P*=.002; Cohen *d*=0.58). This transient deterioration was mainly driven by increased fatigue, pain, insomnia, appetite loss, and reduced physical, role, and social functioning, with social functioning most affected. Emotional and cognitive functioning remained stable. From 3 months onward, HRQoL and all functional domains recovered to baseline or higher levels, with no clinically relevant impairments at 12 months. Symptom burden normalized across most domains after 3 months, although mild constipation remained slightly elevated. Depression scores showed a transient increase at 1 and 3 months but remained below clinical thresholds, whereas anxiety scores were stable at all time points. In univariate regression analyses, no clinical or functional parameters were identified as independent predictors of HRQoL. Overall, recovery was rapid, long-term HRQoL was preserved, and treatment-related morbidity remained low.

**Conclusions:**

Focal HIFU hemiablation preserves long-term HRQoL and psychological well-being in carefully selected patients with localized PCa. Early postoperative impairment is temporary, with recovery to baseline and convergence with normative population values from 3 months onward. Clinical and functional parameters were not significant predictors of HRQoL. These findings support focal HIFU as a minimally invasive quality-of-life–preserving treatment option.

## Introduction

Prostate cancer (PCa) is one of the most commonly diagnosed malignancies among men worldwide and represents a major global health burden [1-3]. Although radical prostatectomy (RP) and radiotherapy (RT) provide excellent oncological control, both are associated with substantial risks of urinary incontinence and erectile dysfunction, which can significantly impair health-related quality of life (HRQoL) [4-7]. Concurrently, widespread early detection has increased the diagnosis of low-risk and potentially indolent tumors, intensifying concerns about overtreatment [8-10].

Active surveillance (AS) is an established strategy to avoid unnecessary intervention in low-risk PCa. However, it is limited by uncertainties in tumor staging and biological behavior. In addition, the psychological burden of living with an untreated malignancy leads up to 20% of patients to choose definitive treatment despite low oncological risk, and approximately one-third discontinue AS within 2 years [11,12]. Repeated prostate biopsies may further impair erectile function, prompting some eligible patients to pursue radical therapy [13,14].

These limitations highlight the need for treatment approaches that preserve oncological safety while minimizing functional and psychological morbidity. In this context, focal therapy has emerged as a promising alternative, aiming to eradicate the index lesion while sparing uninvolved prostate tissue. High-intensity focused ultrasound (HIFU) is one of the most extensively studied focal modalities and has been shown to reduce treatment-related side effects compared with whole-gland approaches [15-20]. Hemiablation—often referred to as “male lumpectomy”—provides lobe-directed tumor control in carefully selected patients [21-24]. Accurate tumor localization is essential for safe and effective focal therapy planning and is optimally achieved using multiparametric magnetic resonance imaging (mpMRI) combined with systematic and/or targeted biopsy [25-29].

Across all PCa treatment strategies, preservation of HRQoL has become a central outcome measure and an important component of shared decision-making. Quality of life, originally introduced as a sociopolitical concept in the 1960s [30,31], is defined by the World Health Organization as a subjective, multidimensional construct encompassing physical, psychological, social, and environmental domains [32]. Owing to its personal and nondirectly measurable nature, HRQoL assessment relies on validated patient-reported outcome measures [33,34].

In oncology, HRQoL reflects a paradigm shift toward a biopsychosocial understanding of disease and plays a growing role in clinical evaluation, health-economic decision-making, and outcomes research [35,36]. Given that oncological success may be offset by functional impairments, HRQoL often becomes a decisive factor in individual treatment selection [37]. Despite the increasing adoption of focal HIFU, prospective data on HRQoL and psychological outcomes following hemiablation remain limited.

This study therefore aimed to evaluate longitudinal changes in HRQoL and psychological distress in patients undergoing focal HIFU hemiablation for unilateral PCa who met the criteria for AS but opted for focal therapy after shared decision-making. In particular, this study examined short-term and 12-month patient-reported outcomes to assess whether focal HIFU preserves quality of life and psychological well-being in this carefully selected patient population.

## Methods

### Patient Cohort and Study Design

This study represents a secondary analysis of prospectively collected patient-reported outcome data from the multicenter HEMI study by Ganzer et al [38], a prospective single-arm clinical trial evaluating focal HIFU hemiablation in men with unilateral, organ-confined PCa. Key functional and oncological outcomes were predefined primary endpoints of the parent HEMI study and have been published previously [38]. The study was registered with the German Working Group on Urological Oncology (AUO) under study protocol AP 68/11.

Patient-reported outcomes, including HRQoL and psychological distress, were prospectively collected according to the study protocol. The present analysis was conducted after the completion of data collection and focuses specifically on longitudinal changes in these outcomes over a 12-month follow-up period.

The study population comprised 54 consecutively enrolled patients who fulfilled the criteria for AS [39] and presented with histologically confirmed unilateral disease and concordant findings on mpMRI. At the time of study conduct, focal therapy was recommended to be performed only within clinical trials. All patients were therefore recruited within the HEMI study after being informed about guideline-conforming treatment options, including AS, RP, and RT, within a shared decision-making process. The final sample size reflects the strictly selected patient population, which applied predefined inclusion and exclusion criteria to identify men suitable for focal hemiablation. All patients were consecutively enrolled at the participating centers during the study period. The present analysis included all patients with available baseline and follow-up patient-reported outcome data. No additional selection or sampling was performed. Therefore, the sample size represents the complete cohort of eligible patients within the study framework. Patients who developed secondary malignancies during follow-up were excluded from longitudinal analyses, as these represent competing health events that would confound the assessment of HRQoL outcomes.Textbox 1

Textbox 1. **Inclusion and exclusion criteria.**

**Inclusion criteria (all must be fulfilled)**

- Male patients aged 18 years and older
- Prostate-specific antigen (PSA) ≤10 ng/mL
- Clinical stage T1c-T2a
- Biopsy-proven unilateral prostate cancer detected by magnetic resonance imaging (MRI) or transrectal ultrasound scan (TRUS) fusion biopsy
- 30% or fewer positive biopsies
- Gleason score of 3+4=7 or less
- Peripheral zone height to be 30 mm or less and 40 mm or less on TRUS in patients treated with Ablatherm integrated imaging and the focal one device

**Exclusion criteria (excluded if *any* of the following criteria applied)**

- Significant cancer of the contralateral side on multiparametric MRI as defined by a score of 4 or greater on PIRADS (Prostate Imaging Reporting and Data System)
- Previous prostatic or urethral surgery
- Intake of 5-*α* reductase inhibitor
- Previous androgen deprivation therapy
- Incomplete clinical or imaging data (eg, missing PSA)

The therapeutic strategy and technical execution of focal HIFU therapy followed the standardized protocol described by Ganzer et al [38], which served as the clinical reference framework for the present analysis. Baseline assessments were performed on the day prior to treatment. Patient enrollment took place between April 2013 and March 2016, corresponding to a recruitment period of 2 years and 11 months. Follow-up evaluations were conducted at 1, 3, 6, and 9 months post intervention using standardized mailed questionnaires. The final 12-month assessment was performed in conjunction with a scheduled rebiopsy at the study center. Time was included as a fixed effect in the longitudinal models without specification of random slopes. The study protocol was initially approved by the institutional review board of the University of Regensburg (Ethics Committee reference: 11-101-0156; approval date: December 29, 2011) and thereafter by the other participating study centers. All participants provided written informed consent prior to inclusion. The inclusion and exclusion criteria applied during study selection are summarized in Textbox 1.

### HRQoL Assessment (EORTC QLQ-C30)

HRQoL was assessed using the European Organization for Research and Treatment of Cancer Quality of Life Questionnaire—Core 30 (EORTC QLQ-C30, version 3.0), a validated and internationally established instrument extensively used in oncological research [40]. The questionnaire comprises 30 items assessing:

- Global health status and quality of life
- Five functional domains (physical, role, emotional, cognitive, and social)
- Three multi-item symptom scales (fatigue, pain, and nausea and vomiting)
- Six single-item symptom dimensions (dyspnea, insomnia, appetite loss, constipation, diarrhea, and financial difficulties)

Items 1 to 28 are rated on a 4-point Likert scale, ranging from “not at all” to “very much,” whereas items 29 and 30 are scored on a 7-point scale assessing global health and overall quality of life.

All scales were scored and transformed according to the EORTC scoring manual [41], resulting in standardized scores ranging from 0 to 100. Higher scores on global and functional scales reflect better functioning and quality of life, whereas higher scores on symptom scales indicate greater symptom burden (Table 1). Raw scores were calculated as the arithmetic mean of completed items per scale and subsequently linearly transformed based on each scale’s scoring range.

Longitudinal changes in HRQoL were analyzed using linear mixed-effects models with random intercepts for participants to account for within-patient correlations over time. Time was included as a fixed effect. Analyses were conducted using restricted maximum likelihood estimation. To control for type I error inflation resulting from multiple testing, false discovery rate (FDR) adjustment according to Benjamini-Hochberg was applied.

**Table 1.** Evaluation of global health status and quality of life, functional scales, general symptom and problem scales, and symptom scales.

Calculated scale symptoms score	Global health status and functional scales	Symptom scales
100	No impairment	Severe symptoms
76‐99	Slight impairment	Moderate to severe symptoms
51‐75	Mild impairment	Moderate symptoms
26‐50	Moderate impairment	Few symptoms
1‐25	Moderate to severe impairment	Slight symptoms
0	Severe impairment	No symptoms

### Comparison With Normative Reference Data and Interpretation of Clinical Relevance

EORTC QLQ-C30 scores of the study cohort were compared with male normative reference values from 2 population-based datasets of the EORTC validation program: the German cohort reported by Schwarz and Hinz and the Norwegian reference population published by Hjermstad et al [42,43]. Inferential analyses were performed exclusively using the German normative dataset, while Norwegian reference values were used for descriptive comparison only. Statistical comparisons with the German population were conducted using Welch two-sided *t* test, with *P* values adjusted for multiple testing using the Benjamini-Hochberg FDR procedure.

Clinical relevance was interpreted according to the classification system proposed by Osoba et al [44], which defines mean score differences of 5 to 10 points as small, 10 to 20 points as moderate, and >20 points as clinically large. As these thresholds were originally derived from breast and lung cancer populations, their applicability to PCa should be interpreted with caution. Only differences meeting predefined criteria for clinical relevance were included in the comparative summary table. All comparisons with normative populations were performed without adjustment for age or comorbidity and are therefore interpreted descriptively rather than causally.

### Psychological Assessment

Psychological distress was assessed using the Hospital Anxiety and Depression Scale (HADS), a validated self-report instrument specifically developed for use in medically ill populations [45,46]. The questionnaire comprises 14 items, with 7 items each evaluating anxiety and depressive symptoms. Each item is rated on a 4-point scale (0‐3), yielding subscale scores ranging from 0 to 21. Clinically relevant cutoff values were set at ≥11, in accordance with published validation studies [47]. Scoring followed the recommendations of the official HADS manual.

### Statistical Analysis

All questionnaire data were entered into an electronic database and analyzed using DATAtab (Seiersberg, Austria). Continuous variables are reported as mean (SD). Although individual questionnaire items are ordinal in nature, aggregated scale and subscale scores were treated as approximately interval-level variables to enable parametric statistical analyses.

Longitudinal changes in HRQoL were analyzed using linear mixed-effects models with random intercepts for participants to account for within-patient correlations over time. Time was included as a fixed effect, and analyses were conducted using restricted maximum likelihood estimation. Comparisons between the study cohort and normative reference populations were conducted using Welch *t* test to account for unequal variances. To control for type I error inflation resulting from multiple testing, FDR adjustment according to Benjamini-Hochberg was applied to all longitudinal analyses and normative comparisons across EORTC QLQ-C30 scales and time points. All statistical tests were 2-sided, and statistical significance was defined as *P*<.05.

Receiver operating characteristic (ROC) curve analysis was performed to assess the discriminatory ability of baseline clinical and functional parameters for HRQoL at 12 months. The EORTC QLQ-C30 global health score was dichotomized at the median. Univariate ROC curves were calculated, and the area under the curve (AUC) was used as a measure of discrimination. ROC curves were visually smoothed for presentation.

### Auditing, Data Monitoring, and Confidentiality

Semiannual audits and monitoring were conducted for data oversight at the study center. These procedures ensured data quality, protocol compliance, and proper conduct of the trial. Participant data were stored in a password-protected database accessible only to authorized study investigators. All personal information was handled in accordance with data protection regulations to ensure confidentiality. Within the framework of the study, patient insurance was in place to cover both the treatment and the entire follow-up period.

### Ethical Considerations

The submitted manuscript meets all criteria of the ICMJE (International Committee of Medical Journal Editors) recommendations and COPE (Committee on Publication Ethics) guidelines. All procedures performed in this study were conducted in accordance with the ethical standards outlined in the 1964 Declaration of Helsinki and its later amendments or comparable ethical standards of the institutional and national research committees. Ethical approval was granted by the Ethics Committee of the Medical Faculty of the University of Regensburg (Ethics Committee reference: 11-101-0156; approval date: December 29, 2011) and subsequently by the ethics committees of the other participating study centers. The committees confirmed that there were no ethical or scientific concerns. Written informed consent was obtained from all participants prior to their inclusion in the study. All data were anonymized, and no information enabling individual identification is presented in this manuscript.

## Results

### Study Population and Follow-Up

**Figure 1.**
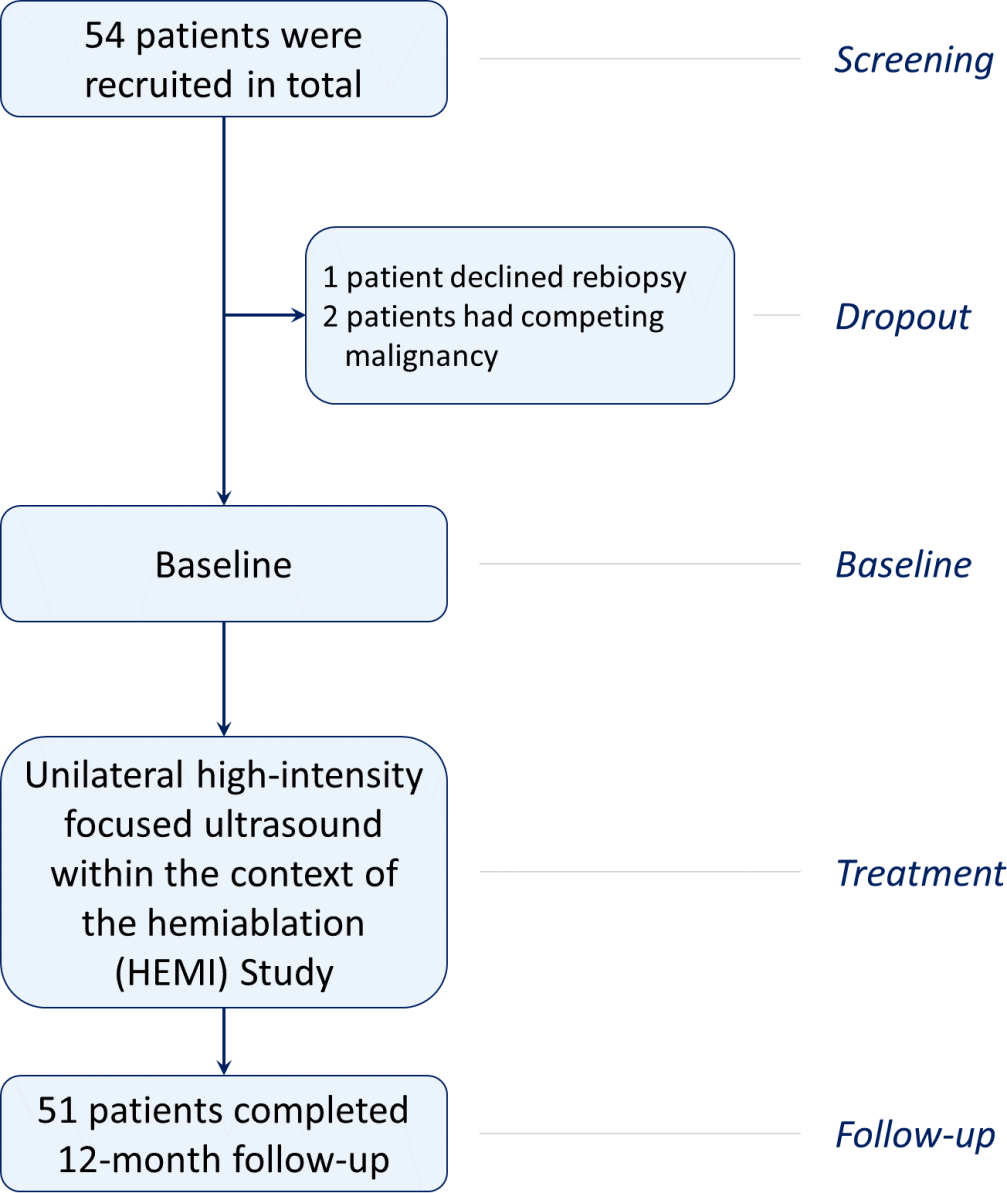
CONSORT (Consolidated Standards of Reporting Trials)-style flow diagram illustrating patient selection.

Fifty-four patients were included in the analysis. Of these, 51 (94.4%) completed the planned 12-month assessment and were included in the final analysis. One patient declined the scheduled rebiopsy, and 2 patients discontinued follow-up due to secondary malignancies. Ultimately, data from 51 patients were available for the final analysis. The study selection process is illustrated in the CONSORT (Consolidated Standards of Reporting Trials) flow diagram (Figure 1). The mean age at treatment was 63.4 (SD 8.3) years, reflecting a typical PCa patient cohort, predominantly comprising a middle-aged population considered suitable for focal therapy. Patient characteristics are summarized in Table 2. Hemi-HIFU was associated with a significant but partial decline in erectile function, while urinary continence and lower urinary tract symptoms were largely preserved. These functional outcomes constituted the primary endpoints of the original study by Ganzer et al [38].

**Table 2.** Patients' characteristics.

	All patients (N=51)
Age (y), mean (SD)	63.4 (8.3)
PSA^a^ (ng/mL), median (IQR)	6.1 (5.1‐7.9)
Number of positive cores, median (IQR)	2 (1-2)
Prostate volume (mL), median (IQR)	32 (27‐41)
Body mass index (kg/m^2^), mean (SD)	28.6 (4.3)
ISUP 2^b^, n (%)	8 (15.7)

a PSA: prostate-specific antigen.

b ISUP 2: International Society of Urological Pathology Grade 2.

### Health-Related Quality of Life (EORTC QLQ-C30)

#### Global Health Status and Quality of Life

**Figure 2.**
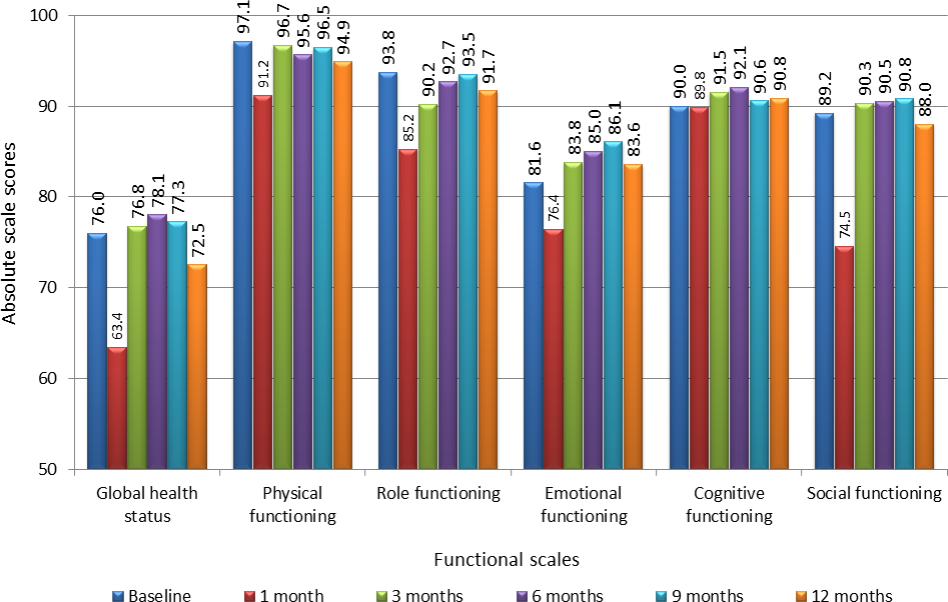
Overview of the absolute mean scale scores for global health status and overall quality of life and the functional parameters across the entire observation period. Higher scores reflect better functioning and a higher quality of life.

At baseline, the mean global health status was 75 (SD 17.8), indicating only minor impairment. At 1 month posttreatment, a significant decline was observed (mean 63, SD 17.9; FDR-adjusted *P*=.002), corresponding to a clinically moderate deterioration. From 3 months onward, scores recovered to baseline or higher levels (3 mo: 78; 6 mo: 78; 9 mo: 77; and 12 mo: 74). Scores at 3 to 9 months were significantly higher than at 1 month (all FDR-adjusted *P=.*004, *P=*.003, and *P=*.004). By month 12, no significant difference from baseline was detected (FDR-adjusted *P*=.39), indicating full recovery of global quality of life (Figure 2; Table 3). The decline in global health status at 1 month corresponded to a medium effect size (Cohen *d*=0.58), and recovery between 1 and 3 months showed a comparable magnitude (*d*=0.61).

**Table 3.** A comprehensive overview of all scores and the corresponding *P* values.

Variable	Baseline	1 month	3 months	6 months	9 months	12 months
Global health status and quality of life, mean (SD)	74.6 (17.8)	63.4 (17.9)	77.5 (18.2)	78.0 (18.8)	77.5 (21.0)	73.9 (22.0)
*P* value	—^a^	.002^b^	.38	.31	.44	.39
Physical functioning, mean (SD)	97.1 (9.0)	91.2 (18.2)	96.7 (7.5)	95.6 (11.1)	96.5 (8.7)	94.4 (10.6)
*P* value	—	.004^b^	.38	.10	.45	.05
Role functioning, mean (SD)	93.8 (17.9)	85.2 (21.3)	90.2 (22.3)	92.7 (20.1)	93.5 (18.8)	91.7 (19.6)
*P* value	—	.04^b^	.43	.57	.72	.29
Emotional functioning, mean (SD)	81.6 (19.8)	76.4 (22.0)	83.8 (20.3)	85.0 (19.3)	86.1 (17.7)	83.6 (21.4)
*P* value	—	.20	.58	.38	.24	.63
Cognitive functioning, mean (SD)	90.0 (15.5)	89.8 (16.0)	91.5 (15.1)	92.1 (15.4)	90.6 (16.8)	90.8 (14.6)
*P* value	—	.76	.21	.67	.84	.82
Social functioning, mean (SD)	89.2 (17.0)	74.5 (25.0)	90.3 (16.9)	90.5 (18.1)	90.8 (19.0)	88.0 (20.2)
*P* value	—	<.001^b^	.74	.69	.60	.73
Fatigue, mean (SD)	13.7 (22.6)	28.5 (30.4)	13.4 (20.2)	12.9 (20.4)	12.5 (19.5)	14.7 (21.8)
*P* value	—	.007^b^	.94	.86	.78	.82
Nausea and vomiting, mean (SD)	2.1 (9.1)	0.9 (3.8)	0.3 (2.1)	1.4 (4.6)	0.6 (3.0)	1.3 (4.4)
*P* value	—	.66	.18	.66	.32	.71
Pain, mean (SD)	5.2 (17.7)	16.2 (17.7)	7.8 (18.5)	11.0 (22.7)	8.2 (19.3)	11.5 (20.0)
*P* value	—	.008^b^	.14	.03^b^	.053	.06
Dyspnea, mean (SD)	5.2 (16.3)	5.6 (14.9)	5.9 (16.5)	5.9 (15.3)	6.2 (15.6)	10.6 (15.2)
*P* value	—	.34	.08	.32	.53	.06
Insomnia, mean (SD)	17.7 (26.1)	29.6 (32.1)	17.5 (26.9)	14.7 (24.0)	15.5 (25.9)	18.0 (26.9)
*P* value	—	.04^b^	.65	.25	.11	.19
Appetite loss, mean (SD)	2.4 (8.7)	6.5 (19.0)	1.1 (6.0)	2.2 (8.3)	2.9 (11.3)	2.3 (8.2)
*P* value	—	.002^b^	.16	.56	.26	.89
Constipation, mean (SD)	4.2 (14.4)	13.5 (25.4)	4.8 (15.3)	4.4 (12.5)	4.0 (12.1)	6.1 (14.3)
*P* value	—	.16	.72	.59	.59	.83
Diarrhea, mean (SD)	5.4 (17.9)	8.1 (21.4)	6.4 (15.9)	6.6 (21.5)	8.0 (19.6)	4.2 (10.4)
*P* value	—	.07	.56	.30	.23	.33
Financial difficulties, mean (SD)	4.2 (14.7)	8.1 (20.1)	3.8 (10.6)	4.4 (13.1)	3.5 (12.4)	4.9 (11.9)
*P* value	—	.10	.17	.59	.32	.47
Depression, mean (SD)	8.8 (4.7)	10.8 (1.9)	10.7 (2.1)	10.6 (2.5)	10.4 (3.0)	10.1 (3.2)
*P* value	—	.02^b^	.02^b^	.07	.23	.28
Anxiety, mean (SD)	6.5 (3.8)	7.8 (1.8)	7.5 (1.7)	6.8 (2.0)	7.0 (1.7)	6.8 (1.8)
*P* value	—	.30	.43	.70	.81	.80

a Not applicable.

b Statistically significant values.

### Functional Scales

#### Physical, Role, Emotional, Cognitive, and Social Functioning

At baseline, physical, role, and cognitive functioning were high (means ≥90, SD 9 and 15), while emotional and social functioning were slightly lower (means 89, SD 19 and 82, SD 17, respectively). At 1 month, physical, role, and social functioning showed transient deterioration, with social functioning demonstrating the most pronounced decline (mean reduction of 14 points, from 89 to 75, SD 25; *P*=.004, *P*=.04, and *P*<.001, respectively). Emotional and cognitive functioning remained stable throughout. From 3 months onward, all functional domains improved. By month 12, no significant differences from baseline persisted across any functional scale, and clinically relevant impairments were rare (Figure 2; Table 3).

### Symptom Scales—General Symptoms and Problem Burden

#### Fatigue, Nausea and Vomiting, Pain, Dyspnea, Insomnia, Appetite Loss, Constipation, Diarrhea, and Financial Difficulties

Baseline symptom burden was low across all domains. At 1 month, fatigue, pain, and insomnia increased transiently. Fatigue (mean increase of 15 points, SD 8.5; *P*=.007), insomnia (mean increase of 12 points, SD 6.9; *P*=.04), and pain (mean increase of 11 points, SD 4.4; *P*=.008) reached clinically moderate severity, while all other symptoms remained in the mild range.

**Figure 3.**
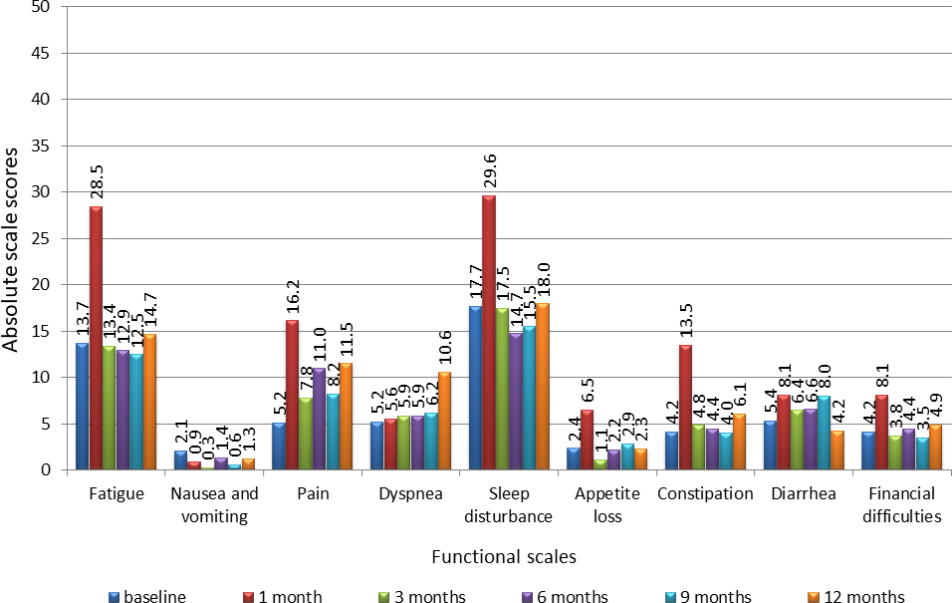
Overview of the absolute mean scale scores for the symptom scales across the entire observation period. Higher scores indicate a greater symptom burden.

From 3 months onward, symptoms normalized across all domains except for pain. In this domain, scores remained significantly different from baseline at 6 months (*P*=.03), but no differences compared to baseline were observed at 9 or 12 months. At no time point was the cumulative symptom burden suggestive of persistent treatment-related morbidity (Figure 3; Table 3).

### HADS—Depression

**Figure 4.**
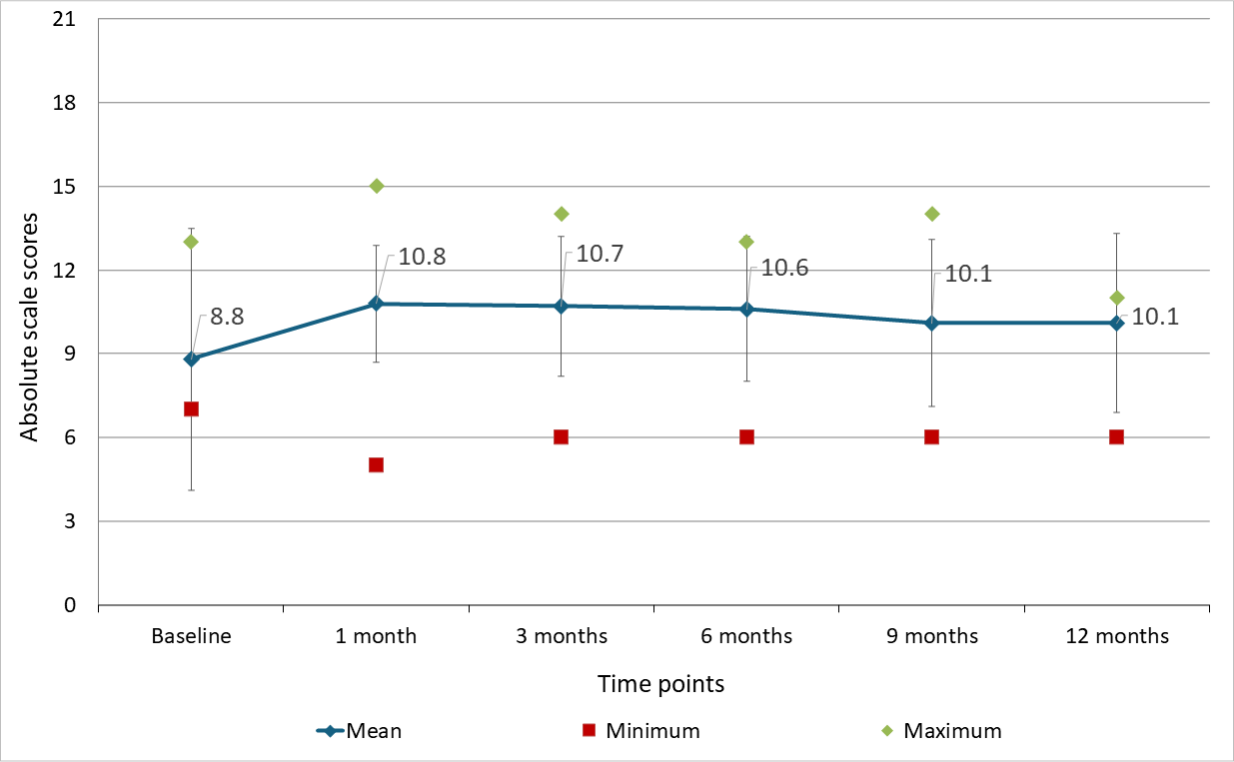
Hospital Anxiety and Depression Scale depression scores over time.

Mean depression scores remained below the clinical cutoff value (≥11) at all assessment points. Statistically significant changes were observed at 1 and 3 months (*P*=.02 and *P*=.02, respectively), indicating a transient but statistically detectable worsening in depressive symptom burden. From the 6-month assessment onward, no differences compared with baseline were detected (all FDR-adjusted *P*>.05 for pairwise comparisons). These findings indicate that clinically relevant depressive symptoms were not present at baseline, increased significantly at 1 and 3 months without exceeding the clinical threshold, and subsequently returned to baseline levels and remained stable throughout follow-up, suggesting recovery from a short-term postinterventional effect rather than persistent psychological deterioration attributable to HIFU therapy (Figure 4; Table 3).

### HADS-D—Anxiety

**Figure 5.**
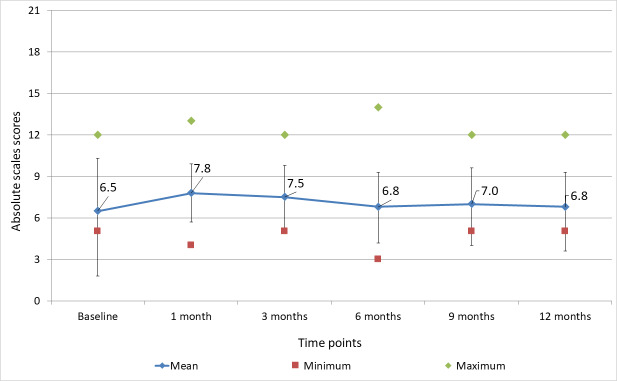
Hospital Anxiety and Depression Scale (HADS) anxiety scores over time.

Mean anxiety scores remained below the threshold throughout, with no significant changes over time (FDR-adjusted *P*>.05). The proportion of patients within the nonpathological range increased slightly during follow-up (Figure 5; Table 3).

An overview of all scale scores from both the functional and symptom domains of the EORTC QLQ-C30, as well as HADS values, including all baseline-referenced *P* values, is presented in Table 3.

Exploratory subgroup analyses comparing patients with and without histologically confirmed recurrence at 12 months revealed no statistically significant differences in any EORTC QLQ-C30 domain or HADS subscale (all *P*>.05). No clinically relevant differences in anxiety or depression scores were observed between the groups.

### Exploratory ROC Analysis of Predictors of 12-Month Global Quality of Life

**Figure 6.**
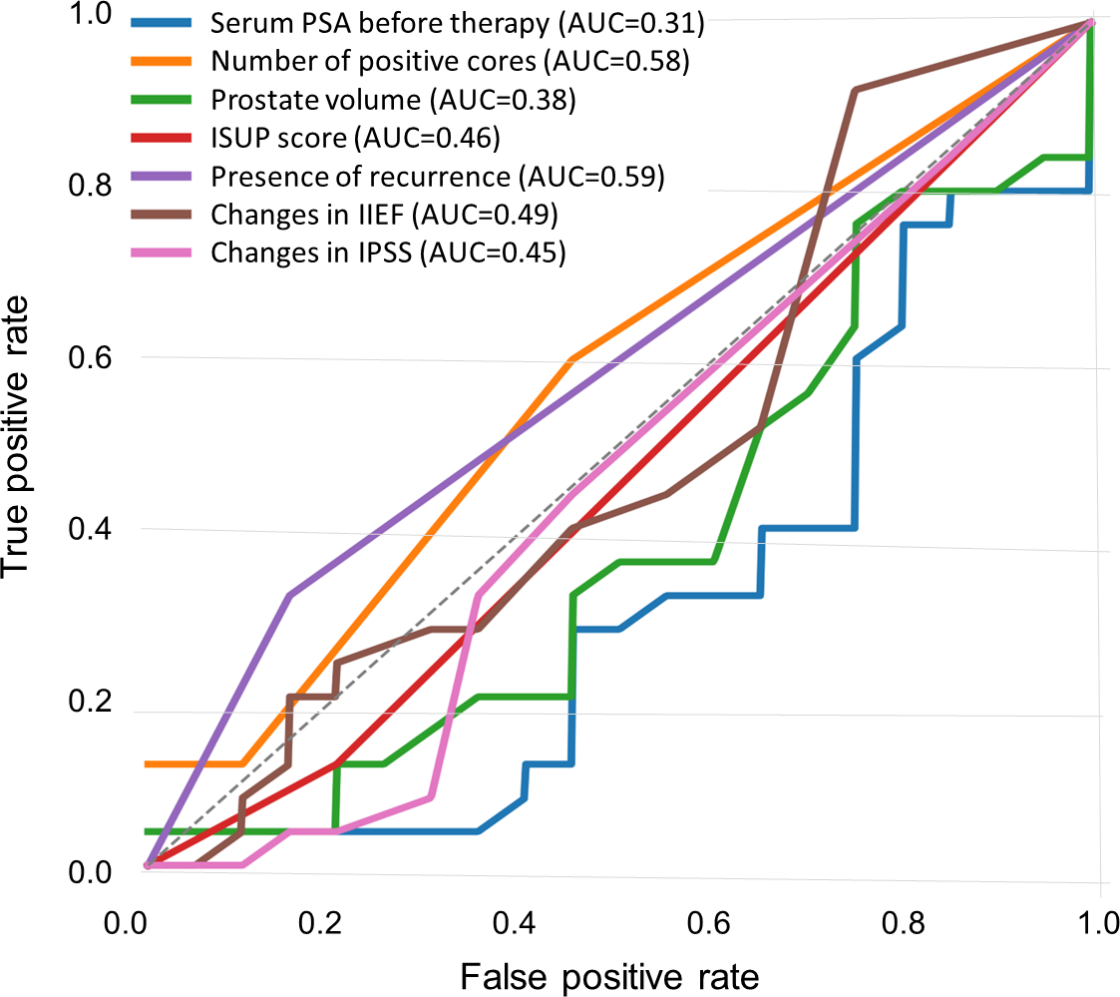
Receiver operating characteristic curves illustrating the discriminatory performance of clinical parameters for predicting European Organization for Research and Treatment of Cancer Quality of Life Questionnaire—Core 30 (EORTC QLQ-C30) scores at 12 months. Univariate models were constructed for prostate volume, serum prostate-specific antigen (PSA), number of positive cores, International Society of Urological Pathology (ISUP) score, presence of recurrence, changes in IPSS (International Prostate Symptom Score), and changes in IIEF (International Index of Erectile Function). The dashed diagonal line indicates no discriminative ability (AUC=0.5). AUC: area under the curve.

To further explore the discriminatory ability of selected clinical and functional parameters for 12-month global HRQoL, univariate ROC curve analyses were performed using dichotomized EORTC QLQ-C30 global health scores at 12 months. Overall, discriminatory performance was low across all variables. The highest AUC values were observed for the presence of recurrence (AUC=0.59) and the number of positive biopsy cores (AUC=0.58), indicating only weak predictive ability. Serum prostate-specific antigen before therapy showed an AUC of 0.31, suggesting an inverse association with the outcome. Prostate volume (AUC=0.38), International Society of Urological Pathology score (AUC=0.46), changes in IIEF (International Index of Erectile Function, AUC=0.49), and changes in IPSS (International Prostate Symptom Score, AUC=0.45) also demonstrated poor discrimination. Overall, none of the analyzed parameters showed clinically meaningful predictive performance for 12-month global quality of life (Figure 6).

### Comparisons of the Individual Dimensions With the General Population

**Figure 7.**
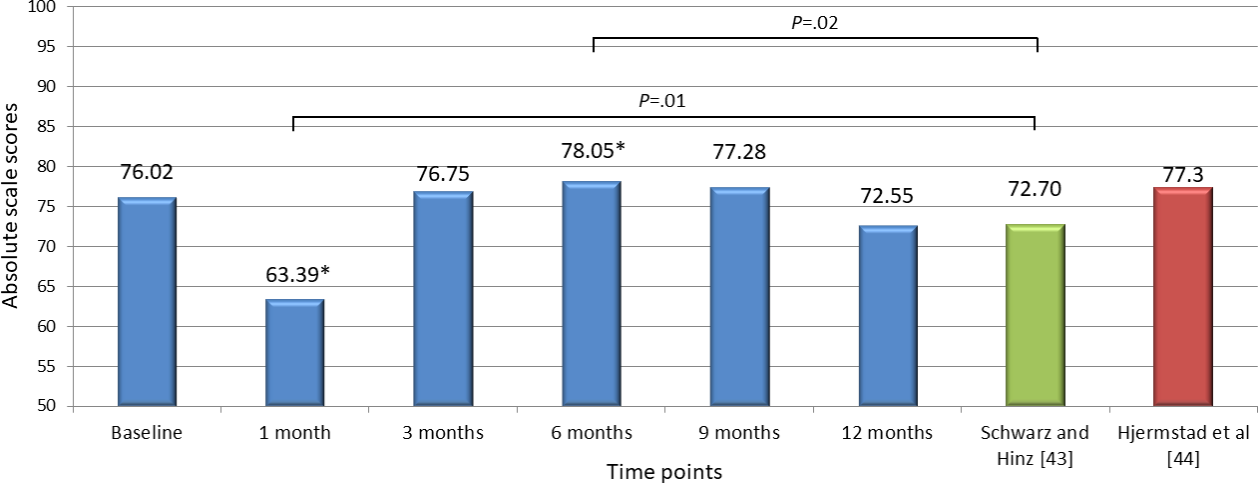
Global health status of the European Organization for Research and Treatment of Cancer Quality of Life Questionnaire—Core 30 (EORTC QLQ-C30) in comparison with the German male normative population. The asterisk (*) indicates values that differ significantly.

**Figure 8.**
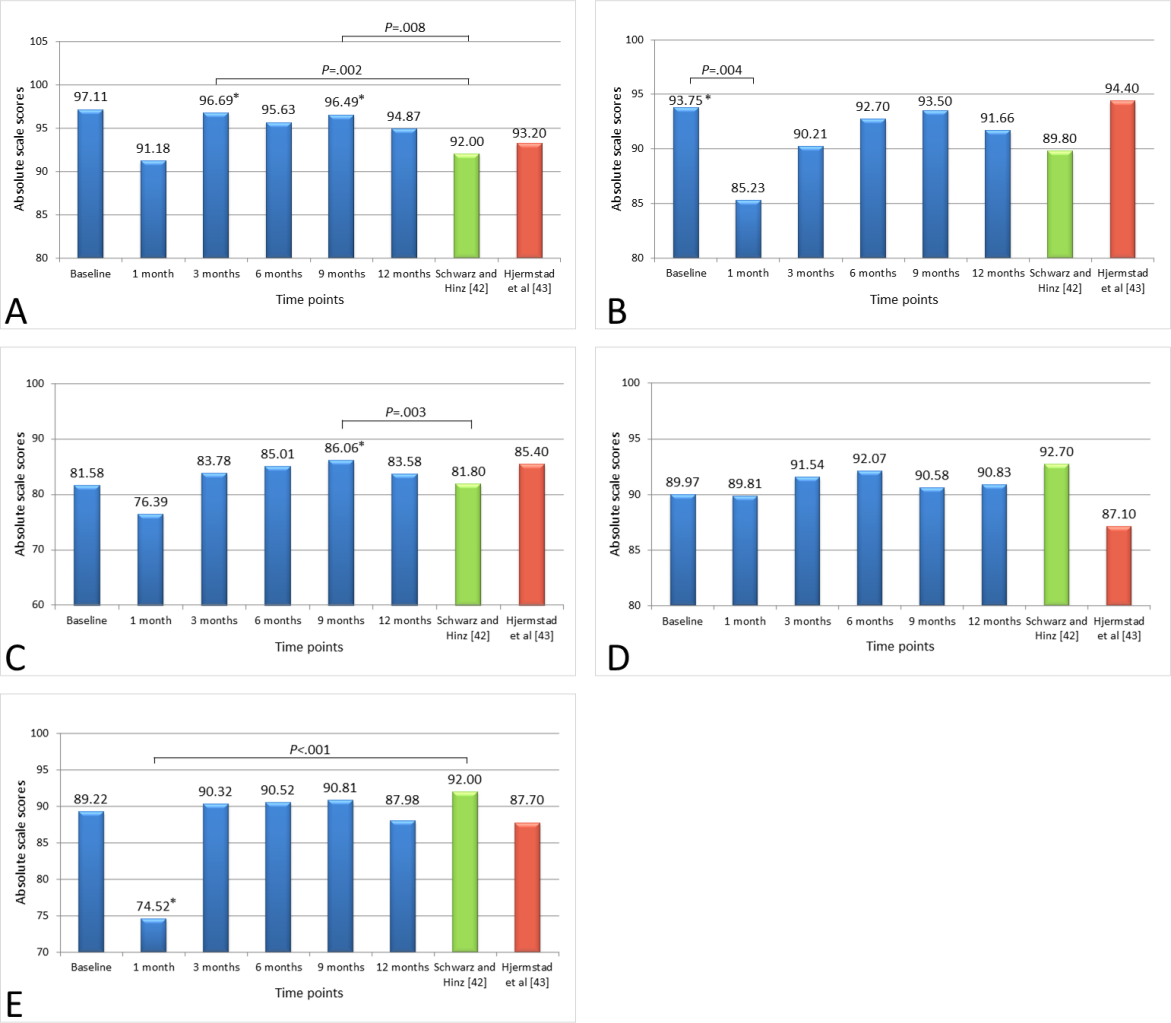
Functional scales of the European Organization for Research and Treatment of Cancer Quality of Life Questionnaire—Core 30 (EORTC QLQ-C30): physical (A), role (B), emotional (C), cognitive (D), and social functioning (E) in comparison with the German male normative population. The asterisk (*) indicates values that differ significantly.

**Figure 9.**
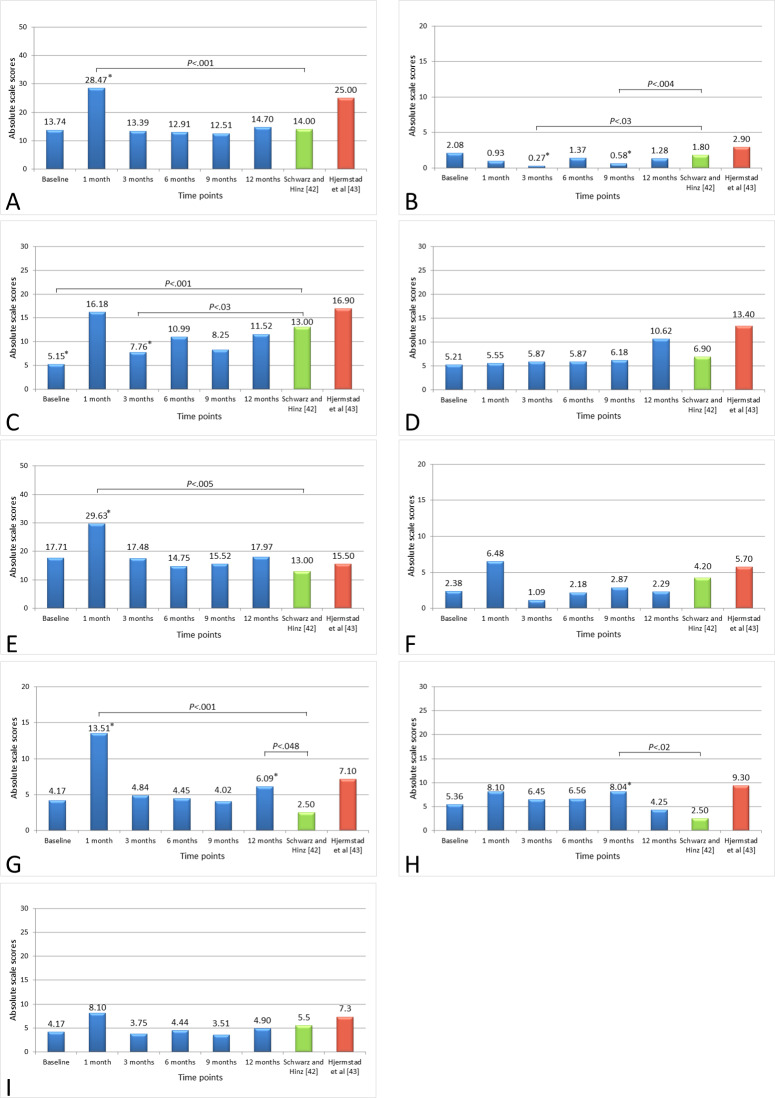
Symptom scales of the European Organization for Research and Treatment of Cancer Quality of Life Questionnaire—Core 30 (EORTC QLQ-C30): fatigue (A), nausea and vomiting (B), pain (C), dyspnea (D), insomnia (E), appetite loss (F), constipation (G), diarrhea (H), and financial difficulties (I) in comparison with male normative population. The asterisk (*) indicates values that differ significantly.

The present analysis compares EORTC QLQ-C30 scores of the study cohort with normative data from healthy male populations. Reference values were obtained from the German general population dataset published by Schwarz and Hinz [42] and from the Norwegian normative dataset reported by Hjermstad et al [43], the latter lacking SD values. Both datasets were originally generated as part of the EORTC validation program to assess the reliability and validity of the QLQ-C30. In the following figures, the study cohort is contrasted with these reference populations (Figure 7; Figure 8; Figure 9). Differences between the cohort and the German normative sample were examined using independent 2-sided *t* tests. To distinguish statistical differences from clinically meaningful differences, the interpretation framework proposed by Osoba et al [44] was applied, defining changes of 5 to 10 points as small, 10 to 20 points as moderate, and >20 points as large clinical differences.

### Global Health Status and Quality of Life

After 1 month, patients reported significantly lower global health compared with the German reference population (*P*=.01), corresponding to a clinically moderate difference according to the Osoba classification. At 6 months, scores exceeded normative values (*P*=.02), although the magnitude of the difference remained clinically small. No clinically relevant deviation was present at 12 months (Figure 7; Table 4).

**Table 4.** Overview of functional and symptom scales showing clinical significance according to the Osoba criteria at each assessment time point.

Scale and time point	Scale score of the patient cohort	Reference group score	Difference between groups	Clinical relevance
Baseline				
Pain	5.15	13.0	7.85	Low
1 month				
Global health status	63.39	72.7	9.31	Low
Physical functioning	97.11	92.0	5.11	Low
Emotional functioning	76.39	81.8	5.41	Low
Social functioning	74.52	92.0	17.48	Moderate
Fatigue	28.47	14.0	14.47	Moderate
Insomnia	29.63	13.0	16.63	Moderate
Constipation	13.51	2.5	11.01	Moderate
Diarrhea	8.9	2.5	6.4	Low
3 months				
Pain	7.76	13.0	5.24	Low
6 months				
Global health status	78.05	72.7	5.35	Low
9 months				
Diarrhea	8.04	2.5	5.54	Low
12 months				
None	—^a^	—	—	—

a Not applicable.

### Functional Subscales

#### Physical, Role, Emotional, Cognitive, and Social Functioning

Physical functioning differed significantly from normative values at 3, 6, and 9 months (all FDR-adjusted *P*=.002, *P*=.03, *P*=.008, respectively); however, all differences remained below the threshold for clinical relevance. A small, clinically relevant advantage of the study cohort was observed at baseline. Role functioning showed a significant decline at 1 month (*P*=.04), while no difference from baseline was observed at 3 months, and values subsequently remained comparable to the normative population across all assessments with no significant differences. Emotional functioning showed a statistically significant improvement at 9 months compared with population data (*P*=.03) but the difference was clinically negligible. Cognitive functioning showed no deviations from reference values at any time point.

Social functioning demonstrated the most pronounced deviation. At 1 month, a moderate clinical deterioration of 15 points relative to the normative population was observed (*P*<.001). From 3 months onward, scores normalized completely (Figure 8; Table 4).

### Symptom Scales—General Symptoms and Problem Burden

#### Fatigue, Nausea and Vomiting, Pain, Dyspnea, Insomnia, Appetite Loss, Constipation, Diarrhea, and Financial Difficulties

Baseline symptom burden was low across all domains compared with the German normative population. At 1 month posttreatment, fatigue increased significantly (items 10, 12, and 18; *P*<.001), corresponding to a clinically moderate deterioration (mean difference of 14 points, from 14 to 28, SD 23). Fatigue scores returned to baseline by 3 months and remained stable thereafter.

Nausea and vomiting (items 14 and 15) differed significantly from normative values at 3 and 9 months in favor of the study cohort (*P*=.004 and *P*=.03, respectively); however, absolute symptom levels remained negligible and were not clinically meaningful (maximum mean score of 2.08, SD 9.1). Pain (items 9 and 19) was significantly lower in the study cohort at baseline and 3 months compared with normative data (*P*=.002 and *P*=.31, respectively), corresponding to small-to-moderate clinical effects.

No significant differences were observed for dyspnea at any time point (all *P*>.05), with all deviations falling below clinical relevance thresholds. Insomnia (item 11) demonstrated a clinically moderate short-term deterioration at 1 month (mean difference of 17, SD 32 points; FDR-adjusted *P*=.005), which fully resolved by month 3.

Appetite loss (item 13) differed significantly from normative values only at 3 months (*P*=.002), but the magnitude of the difference was clinically negligible (<5 points). Constipation (item 16) was significantly elevated at 1 and 12 months (FDR-adjusted *P*=.01 and *P*=.048), representing the only symptom with persistent clinically relevant impairment. Diarrhea (item 17) increased at 9 months (*P*=.02), with small but clinically meaningful differences.

No differences were detected for financial difficulties (item 28). Overall, symptom trajectories were characterized by transient early postoperative worsening—most notably for fatigue and insomnia—followed by recovery by month 3 across most domains. Persistent morbidity was limited to constipation, supporting a favorable tolerability profile of focal HIFU therapy (Figure 9; Table 4).

## Discussion

### Summary of Main Findings

This study evaluated psychological distress and HRQoL in patients undergoing focal HIFU hemiablation for localized PCa over a 12-month follow-up period. The primary objective was to characterize the temporal evolution of patient-reported outcomes from baseline through long-term follow-up. Overall, focal therapy was associated with low levels of psychological distress and preserved HRQoL. A statistically significant but transient decline in HRQoL and selected symptom domains was observed at 1 month, followed by recovery to baseline levels by 3 months. At 12 months, no clinically relevant impairments persisted. Depression scores showed a temporary increase without exceeding clinical thresholds, while anxiety levels remained stable throughout follow-up.

### Contextualization

Across all psychological instruments and subscales, focal therapy demonstrated a favorable profile with respect to quality-of-life preservation. Mean scores consistently indicated a low overall psychological burden, with most patients reporting minimal or no impairment. Notably, no statistically significant differences were observed between baseline and 12-month values in any psychological domain, supporting the assumption that focal therapy is associated with stable long-term psychosocial outcomes [48,49].

The transient decline in HRQoL and psychological well-being observed at 1 month is most likely attributable to procedure-related factors, including peri-interventional stress, temporary functional impairment, and recovery from treatment. By 3 months, baseline values were restored and remained stable thereafter. Application of Osoba criteria suggests that clinically meaningful deterioration was largely confined to the early postoperative phase. Similar transient deteriorations have been described following focal ablative therapies and are attributed to perioperative stressors, including hospitalization, anesthesia, and temporary functional impairment [49]. Recent longitudinal studies using PCa-specific outcome measures (eg, IPSS, IIEF-5, EPIC [Expanded Prostate Cancer Index Composite]) similarly demonstrate a transient decline in urinary and sexual function at 1 month following focal HIFU, with recovery to baseline levels from 3 months onward, supporting the functional safety of this approach [50]. Overall, the early postoperative symptom increase appears primarily related to procedural stress rather than oncological anxiety.

Functional and oncological outcomes of the underlying HEMI study have been reported in detail elsewhere [38]. In brief, focal HIFU hemiablation was associated with preserved urinary continence and largely maintained erectile function, with no increase in incontinence rates and potency retained in the majority of preoperatively potent patients. These findings provide important clinical context for the present analysis and support the interpretation of preserved quality-of-life outcomes.

Comparison with normative population data revealed significant deviations primarily at the early postoperative time point, whereas later assessments showed convergence with population-based reference values. This pattern indicates that focal therapy does not result in sustained impairment relative to the general population. Similar recovery trajectories have been reported in previous studies of focal HIFU and other focal treatment modalities [51-53].

Psychological outcomes in this cohort were characterized by low baseline distress and stable trajectories over time. The predominance of patients with early-stage PCa may partly account for the low prevalence of clinically relevant psychological impairment. The temporary increase in depressive symptoms likely reflects a situational stress response rather than a persistent affective disorder. Importantly, no clinically relevant anxiety or depression was observed throughout follow-up. These findings are consistent with previous reports demonstrating that anxiety and depressive symptoms in patients undergoing focal therapy remain close to population norms and are associated with low levels of psychological burden and decision regret [49,54].

When compared with other established treatment modalities for localized PCa, these findings further support the favorable quality-of-life profile of focal therapy. RP and RT have been consistently associated with higher rates of urinary incontinence and sexual dysfunction, which are key determinants of long-term HRQoL [4-7,37]. In contrast, AS is generally associated with preserved functional outcomes but may be accompanied by an increased psychological burden due to ongoing uncertainty regarding disease progression. In this context, focal HIFU may represent a balanced approach, combining the low psychological burden observed in AS with the benefits of active oncological treatment while maintaining favorable functional outcomes.

Exploratory analyses did not demonstrate an association between short-term oncological outcomes and patient-reported quality of life or psychological distress. No statistically significant or clinically relevant differences in EORTC QLQ-C30 domains or HADS scores were observed between patients with and without histologically confirmed recurrence at 12 months. These findings suggest that short-term oncological outcomes, including the presence of residual or recurrent disease, did not translate into measurable impairments in HRQoL or increased psychological burden within the observation period. This may be explained by the low overall tumor burden and favorable disease characteristics of the study population, as well as structured follow-up and patient counseling within the study setting.

However, these results should be interpreted with caution. The number of recurrence events was limited, and the study was not powered to detect subtle subgroup differences. In addition, the relatively short follow-up period may not capture delayed psychological effects related to oncological uncertainty or disease progression.

Additional factors may include patient selection and individual coping mechanisms. Patients with localized, low-risk disease generally exhibit a lower baseline psychological burden than those with advanced cancer. Furthermore, the active decision to undergo focal therapy may enhance perceived control and facilitate psychological adaptation.

The deliberate decision to undergo focal HIFU may represent an additional psychological factor influencing patient-reported outcomes. By selecting a minimally invasive, organ-preserving treatment approach, patients may experience a greater sense of control and involvement in their care. Such perceptions have been associated with reduced feelings of helplessness and improved psychological adjustment. In this context, previously reported low levels of decision regret following focal therapy further support this interpretation [54].

### Conclusion and Clinical Implications

Focal HIFU hemiablation is associated with preserved psychological well-being and sustained HRQoL in patients with localized PCa. Although transient postoperative distress may occur, recovery is rapid, and long-term outcomes remain comparable to population norms.

Taken together, these findings suggest that psychological stabilization following focal therapy is achievable in the context of preserved coping capacity and patient empowerment. Both confrontation with the diagnosis and postinterventional adjustment appear manageable within this treatment framework, supporting focal therapy as a patient-centered approach that balances disease control with quality-of-life preservation.

From a clinical perspective, routine psycho-oncological follow-up may be most relevant for vulnerable subgroups, whereas broader interventions may not be necessary in psychologically stable patients. Future research should focus on extended longitudinal follow-up, the use of standardized patient-reported outcome measures, integration of systematic psycho-oncological screening, identification of vulnerability profiles, and the parallel assessment of oncological and psychological endpoints.

### Limitations

Several limitations should be considered. The predominance of patients eligible for AS reflects historical indications for focal therapy at the time of study initiation (2013‐2016) and may limit the applicability of the findings to contemporary patient populations with clinically significant disease. Since study initiation, indications for focal therapy have evolved toward carefully selected patients with clinically significant localized disease, including favorable intermediate-risk PCa. At the same time, the use of a low-risk, AS-eligible population allowed for an isolated assessment of treatment-related quality-of-life outcomes with minimal confounding by advanced disease burden and contributed to the early evidence base supporting the broader application of focal therapy. Detailed data on the number of screened patients and reasons for nonparticipation were not systematically recorded due to the study design and shared decision-making process.

First, the sample size was relatively small (N=54), and attrition over the 12-month follow-up reduced statistical power, particularly for subgroup analyses and small effect sizes. The exclusion of patients due to protocol-defined dropouts, including competing health events and incomplete follow-up, may introduce a potential risk of selection bias in the longitudinal analyses. In addition, the highly selected study cohort limits generalizability to the broader PCa population.

Second, patient recruitment occurred between 2013 and 2016, and subsequent advances in imaging, biopsy techniques, and treatment guidelines (eg, European Association of Urology, 2024) may limit direct applicability to contemporary clinical practice. Third, the study lacked a randomized control group, relying instead on external normative reference populations, which restricts causal inference and increases susceptibility to unmeasured confounding.

Fourth, although validated instruments were used, patient-reported outcomes are inherently subjective and may be influenced by response and recall bias. In addition, the use of mailed questionnaires may have introduced additional selection bias. Fifth, clinical relevance was interpreted using the Osoba classification, which was originally developed in breast and lung cancer populations, and its applicability to PCa remains uncertain.

Finally, despite the use of mpMRI and targeted biopsies, focal hemiablation carries a risk of undertreatment due to unrecognized multifocal disease. Detailed oncological outcomes were not assessed, and follow-up was limited to 12 months, precluding the evaluation of late functional or psychological effects.

## Abbreviations

AS: active surveillance
AUC: area under the curve
CONSORT: Consolidated Standards of Reporting Trials
EORTC: European Organization for Research and Treatment of Cancer
FDR: false discovery rate
HADS: Hospital Anxiety and Depression Scale
HIFU: high-intensity focused ultrasound
HRQoL: health-related quality of life
mpMRI: multiparametric magnetic resonance imaging
PCa: prostate cancer
PIRADS: Prostate Imaging Reporting and Data System
QLQ-C30: Quality of Life Questionnaire—Core 30
ROC: receiver operating characteristic
RP: radical prostatectomy
RT: radiotherapy
